# Urethral reconstruction and genitoplasty highlighted in International Brazilian Journal of Urology

**DOI:** 10.1590/S1677-5538.IBJU.2021.04.01

**Published:** 2021-07-20

**Affiliations:** 1 Universidade do Estado de Rio de Janeiro Unidade de Pesquisa Urogenital Rio de JaneiroRJ Brasil Unidade de Pesquisa Urogenital - Universidade do Estado de Rio de Janeiro - Uerj, Rio de Janeiro, RJ, Brasil,; 2 Hospital Federal da Lagoa Rio de JaneiroRJ Brasil Serviço de Urologia, Hospital Federal da Lagoa, Rio de Janeiro, RJ, Brasil

The July-August number of Int Braz J Urol, the 11th under my supervision, presents original contributions with a lot of interesting papers in different fields: Prostate Cancer, Male Infertility, Renal Cell Carcinoma, Urinary Stones, Testicular Cancer, Bladder Cancer, LUTS, Neurogenic Bladder, Pediatric Urology, Basic Research, Interstitial cystitis, female genitoplasty, Urethral strictures and Covid-19 in Urology. The papers came from many different countries such as Brazil, USA, China, UK, Italy, Colombia and India, and as usual the editor´s comment highlights some of them.

In the present issue we present three important papers about urethral reconstruction and genitoplasty. Dr. Kalra and colleagues from India performed in page 829 (1) a nice study about the female urethral stricture disease (FUSD) and have tried to characterize the variable clinical presentation of FUSD, the diagnostic utility of calibration, videourodynamic study(VUDS), and urethroscopy in planning surgical management and concluded that a good caliber of the urethra is not sufficient enough to rule out a significant obstruction due to FUSD. Early urethroplasty provides significantly better outcomes in patients who have failed dilation as a treatment.

Dr. Barroso Jr. and Prado from Brazil described in page 856 (2) a modified technique for urethroplasty with longitudinal urethral incision and concluded in this preliminary report that after four and six months of follow-up, the postoperative outcomes were satisfactory for both patients but further studies involving larger numbers of patients and long-term follow-up are required to evaluate the effectiveness of this method. In a nice study in page 861 Dr. Fernandez and colleagues from Colombia (3) studied a new surgical technique for clitoroplasty, completely preserving corporeal bodies, neurovascular bundles without dismembering the clitoris, in four patients with over a year follow up and concluded that they show a new concept of complete corporeal preservation clitoroplasty (CCPC) without the need of disassembling the corporeal bodies, neurovascular bundle and glans. It stands as a new alternative for feminizing genitoplasty with complete preservation of erectile tissue and no dissection of neurovascular bundle, a very nice study. The editor in chief would like to highlight the following works too:

Dr. Matushita and colleagues from Brazil presented in page 705 (4) a nice systematic review about the role of 68Ga-PSMA PET/CT, a non-invasive diagnostic tool to evaluate prostate cancer (PC) with prostate-specific membrane antigen (PSMA) expression and concluded that 68Ga-PSMA PET provides higher sensitivity and specificity than traditional imaging for prostate cancer.

Dr. Wu and colleagues from China presented in page 733 (5) a nice meta-analysis about the associations of circulating and dietary intake of vitamin D with risk of risk of renal cell carcinoma (RCC) and concluded that higher circulating vitamin D level and higher dietary vitamin D intake both might be associated with a reduced risk of RCC but further high-quality randomized controlled trials are required in the future to confirm this results.

Dr. Maia and colleagues from Brazil presented in page 747 (6) a interesting narrative review about the otorhinolaryngological adverse effects of the main drugs used in urological practice and concluded that most of the drugs used in urological practice have otorhinolaryngological adverse effects. Dizziness was most common, but dry mouth, rhinitis, nasal congestion, epistaxis, hearing loss, tinnitus, and rhinorrhea were also reported.

Dr. Prezotti and colleagues from Brazil presented in page 753 (7) a important original paper about COVID-19 pandemic. The authors evaluated the impact of COVID-19 on clinical and surgical practice, educational activities, health and lifestyle behavior of Brazilian urology residents and concluded that COVID-19 had a massive impact in Brazilian urology residents´ training, health and lifestyle behavior, which may reflect what happened in other medical specialties.

Dr. Alvim and colleagues from USA performed in page 777 (8) an interesting study about the treatment of pT3a renal cell cancer (RCC) to compare the oncological and functional outcomes of patients with pT3a RCC scheduled for partial nephrectomy (PN) and radical nephrectomy (RN) and concluded that there are no evidence that patients scheduled to undergo partial nephrectomy had poorer oncologic outcomes than patients scheduled to undergo radical nephrectomy. In select patients with pT3a renal cell carcinoma in whom partial nephrectomy is deemed feasible by the surgeon, partial nephrectomy should not be discouraged.

Dr. Veiga and colleagues from Brazil performed in page 787 (9) an interesting randomized clinical trial about the parasacral transcutaneous electrical nerve stimulation (TENS) in children of at least four years of age with a diagnosis of pure overactive bladder and concluded that children with overactive bladder who are unable to undergo parasacral TENS treatment three times weekly, the method can be administered successfully at twice-weekly sessions.

Dr. Au and colleagues from UK performed in page 803 (10) an interesting study about the neoadjuvant chemotherapy followed by radical cystectomy (NACRC) in treatment for muscle invasive bladder cancer (MIBC) and show that the treatment rates and factors associated with not receiving NACRC in MIBC patients with lower comorbidity status most likely to be candidates for NACRC.

Dr. Cezarino and colleagues from Brazil performed in page 821 (11) a very nice study about the upper-pole nephrectomy to correct symptomatic duplex systems with poor functioning moieties and the distal ureteral stump syndrome (DUSS) - a late complication of this approach with the objective to show if extended ureteral dissection can prevent DUSS in top-down approach and concluded that upper pole nephrectomy should be performed with extended distal ureteral dissection to prevent ureteral stump complications.

Dr. Wu and colleagues from China performed in page 843 (12) a very nice study about a complex disease – the Interstitial cystitis and concluded that the core genes (CXCL8, CXCL1, IL6) obtained in this study may be potential biomarkers of interstitial cystitis with guiding significance for clinical treatment.

Dr. Sobrinho and colleagues from Brazil performed in page 887 (13) the cover page of the present number of Int Braz J Urol. The authors show a nice experimental report and demonstrated a new option for endourological training, using three-dimensional (3D) printing models of kidneys with macroscopic congenital anomalies (horseshoe and duplicated pelvicalyceal collecting system) and concluded that the use of 3D printed kidney models before endourological procedures for pre-surgical training is feasible and can be done with low-cost materials. The surgeon can train before proposing the appropriate surgical schedule to the patient using the 3D printed kidney.

The Editor-in-chief expects everyone to enjoy reading and for sure better times will come soon.
